# The novel mTOR inhibitor CCI-779 (temsirolimus) induces antiproliferative effects through inhibition of mTOR in Bel-7402 liver cancer cells

**DOI:** 10.1186/1475-2867-13-30

**Published:** 2013-03-28

**Authors:** Shuyu Li, Yan Liang, Manlin Wu, Xiaojing Wang, Haixia Fu, Yuhao Chen, Zhigang Wang

**Affiliations:** 1 College of Life Science, Inner Mongolia University, 010021, Hohhot, P.R. China

**Keywords:** CCI-779 (temsirolimus), mTOR signaling, Cell growth, Liver cancer cell

## Abstract

**Background:**

Liver cancer is one of the most frequent cancers in the world. Targeted therapy of cancer with specific inhibitors is developing and has shown promising antitumor efficacy. CCI-779 (temsirolimus), a specific inhibitor of mTOR (mammalian target of rapamycin), can block the mTOR signaling pathway. Here, we systematically examined the expression of mTOR and its downstream targets in liver cancer cells and normal liver cells, then investigated inhibitory effects of CCI-779 on mTOR signaling pathway and its role in regulating liver cancer cell growth.

**Methods:**

The expression of mTOR and its downstream targets in Bel-7402 liver cancer cells and HL-7702 normal liver cells were examined by western blot. The mTOR specific inhibitor (CCI-779) was used to treat Bel-7402 cells to identify its effects on Bel-7402 cell growth and activity of mTOR signaling pathway *in vitro*. Cell viability tests were performed after the treatment of CCI-779. Western blot was applied to assess the changes of mTOR pathway and flow cytometry was used to analyze cell cycle of Bel-7402 cells after the treatment of CCI-779.

**Results:**

mTOR, p70S6K, S6, and 4EBP1 were overexpressed in Bel-7402 cells compared with HL-7702 cells. Bel-7402 cells were sensitive to CCI-779. The survival rate of the cells treated with CCI-779 over 0.312 μM was significantly different compared with that of control (*P* < 0.05). CCI-779 inhibited the phosphorylation of mTOR (Ser2448), p70S6K (Thr389), S6 (Ser240/244), and 4EBP1 (Thr37/46) in different grades and the expressions of p70S6K, S6, and 4EBP1. As a result, CCI-779 induced a dose-dependent decrease in cell proliferation, G1/S arrest and damage of cell shape.

**Conclusions:**

Taken together, these data showed that CCI-779 can inhibit mTOR signaling and proliferation in Bel-7402 liver cancer cells *in vitro*. It offers a therapeutic intervention through inhibition of mTOR as a potential strategy for liver cancer.

## Background

Liver cancer is the sixth most common cancer worldwide and the third leading cause of cancer-related death; 55% of cases are in China [1]. Rates of liver cancer in men are typically 2 to 4 times higher than in women [2]. One of the current treatments for liver cancer is liver transplantation. Surgical resection of liver cancer is associated with a high rate of recurrence [3], due to occult metastases that might exist in the remnant liver at the time of resection or as a result of the ‘field’ effect in the remnant liver [4]. Thus, new therapeutic strategies are needed due to the poor prognosis of patients with liver cancer. Targeting cancers with specific inhibitors is a developing strategy and has shown promising antitumor efficacy.

Increasing knowledge of the signal transduction pathways of growth factors has implicated them as novel targets for cancer therapy. Mammalian target of rapamycin (mTOR) is an evolutionarily conserved protein kinase that belongs to the phosphatidylinositol kinase-related kinase (PIKK) family and functions as a serine/threonine kinase. Mounting evidence has indicated that mTOR and its downstream targets S6K1 and 4EBP1 are linked to tumorigenesis. These proteins are often overexpressed or mutated in various cancers and promote malignant transformation [5]. Thus, inhibition of mTOR signaling is a promising therapeutic anti-cancer strategy.

Rapamycin, the naturally occurring inhibitor of mTOR, and several recently developed rapamycin analogs (RAD001, CCI-779, AP23576, AZD8055) [6,7] inhibit the growth of cell lines that have been derived from many tumor types *in vitro* and tumor models *in vivo*[8]. CCI-779, a dihydroxymethyl propionic acid ester of rapamycin, or temsirolimus, is an effective inhibitor of mTOR. Several groups have studied its suppression of various cancers [9-11], and others have initiated a phase II study of CCI-779 in breast cancer [12] and glioblastoma multiforme [13]. The clinical function of RAD001 has been studied [14], but the inhibitive effects and therapeutic value of CCI-779 in liver cancer remains unclear.

In this study, we analyzed the differences in mTOR expression between liver cancer and normal liver cells and treated Bel-7402 liver cancer cells with CCI-779 to show mTOR signaling has an important role in liver cancer cell growth regulation. Our data showed the inhibitive effects of CCI-779 on mTOR signaling and liver cancer cell growth. It offers a therapeutic intervention through inhibition of mTOR as a potential strategy for liver cancer.

## Results and Discussion

### CCI-779 inhibits proliferation of Bel-7402 liver cancer cells

**Figure 1:**
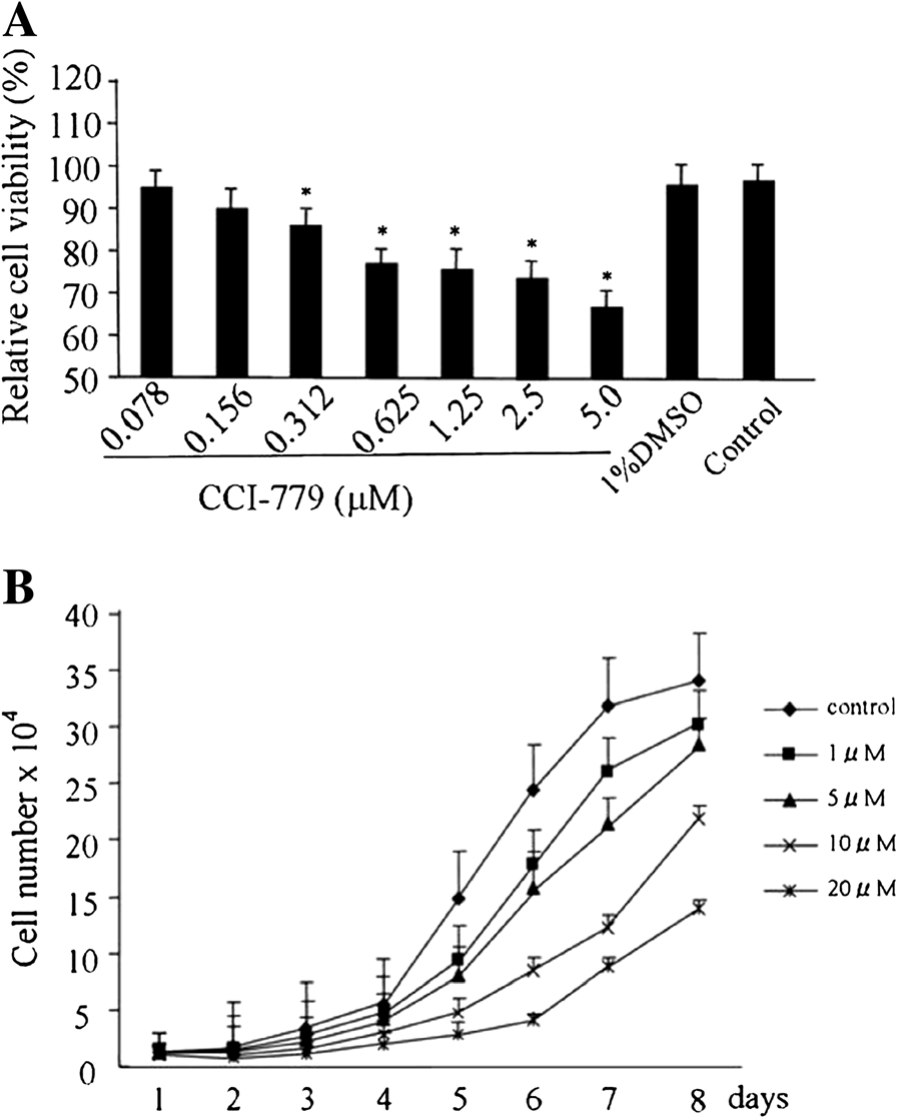
**CCI-779 inhibits proliferation of Bel-7402 liver cancer cells in vitro.** (**A**) The effects of CCI-779 and its solvent DMSO on Bel-7402 cell proliferation were examined by trypan blue exclusion assay. (**B**) CCI-779 treatment on Day 1 and cell number for each condition every 24 h until Day 8. diamond (♦ ), control (DMSO only); rectangle (■), 1 μM of CCI-779; triangle (▲ ), 5 μM CCI-779; fork (×), 10 μM CCI-779; star (✼), 20 μM CCI-779.

The effects of CCI-779 on cell proliferation of Bel-7402 liver cancer cells were examined by trypan blue exclusion assay. Bel-7402 cells were sensitive to CCI-779, and the survival rate of the cells treated with CCI-779 over 0.312 μM was significantly suppressed compared with that of control. There was no significant difference between cells that were treated with 1% DMSO and the control (Figure 1A). As shown in the growth curve in Figure 1B, 1 μM CCI-779 suppressed Bel-7402 growth from 4 to 8 days after treatment.

**Figure 2:**
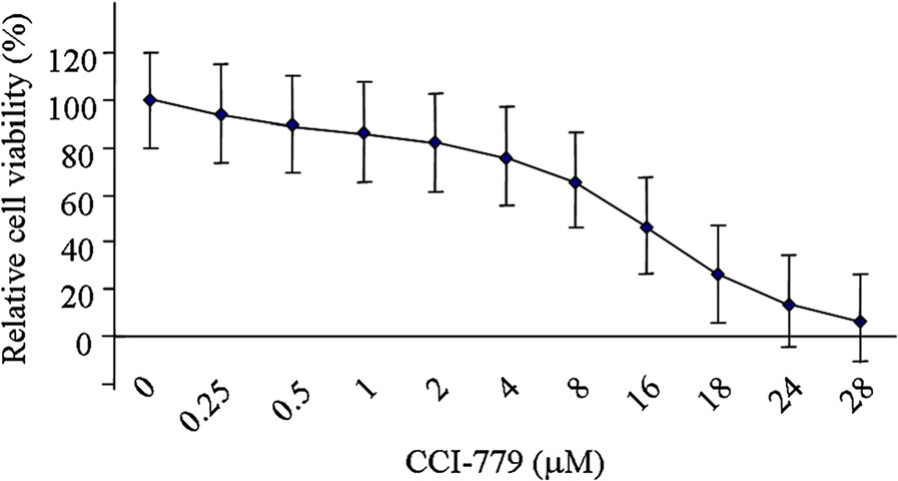
**Inhibition curve of CCI-779 on Bel-7402 growth.** Bel-7402 cells were treated with CCI-779 (0.25-28 μM) for 48 h, and the susceptibility to CCI-779 was determined by MTT assay.

To determine the inhibitory effects of CCI-779 on cell growth and optimize its concentration for subsequent experiments, we calculated the half maximal inhibitory concentration (IC_50_) of CCI-779 in liver cancer cells. Bel-7402 cells were treated with various concentrations of CCI-779 (0.25 μM~28 μM) for 48 h, and their susceptibility to CCI-779 was determined by MTT assay. The IC_50_ of CCI-779 on Bel-7402 cells was 8.62 μM (Figure 2).

### mTOR, p70S6K, S6, and 4EBP1 are overexpressed in Bel-7402 cells

**Figure 3:**
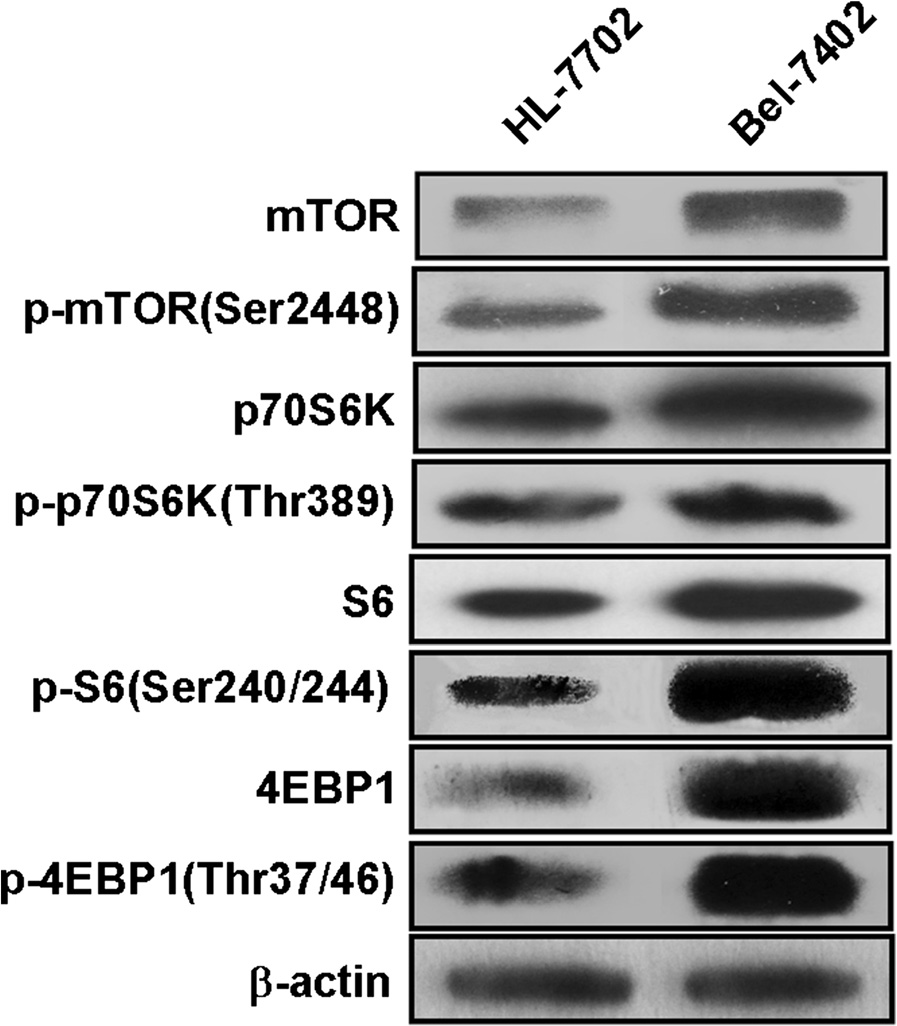
**Expression of mTOR, p70S6K, S6, and 4EBP1 is higher in Bel-7402 cells versus HL-7702 cells.** Expression of mTOR, p70S6K, S6, and 4EBP1 was examined by western blot. β-actin served as gel control.

To examine the expression of mTOR and its downstream targets in Bel-7402 cells, we performed western blot analysis. As shown in Figure 3, the expression of mTOR, p70S6K, S6, and 4EBP1 was higher in Bel-7402 cells than in HL-7702 cells. The phosphorylation of these signaling proteins was also greater in Bel-7402 versus HL-7702 cells.

### CCI-779 inhibits activation of mTOR and its downstream targets

**Figure 4:**
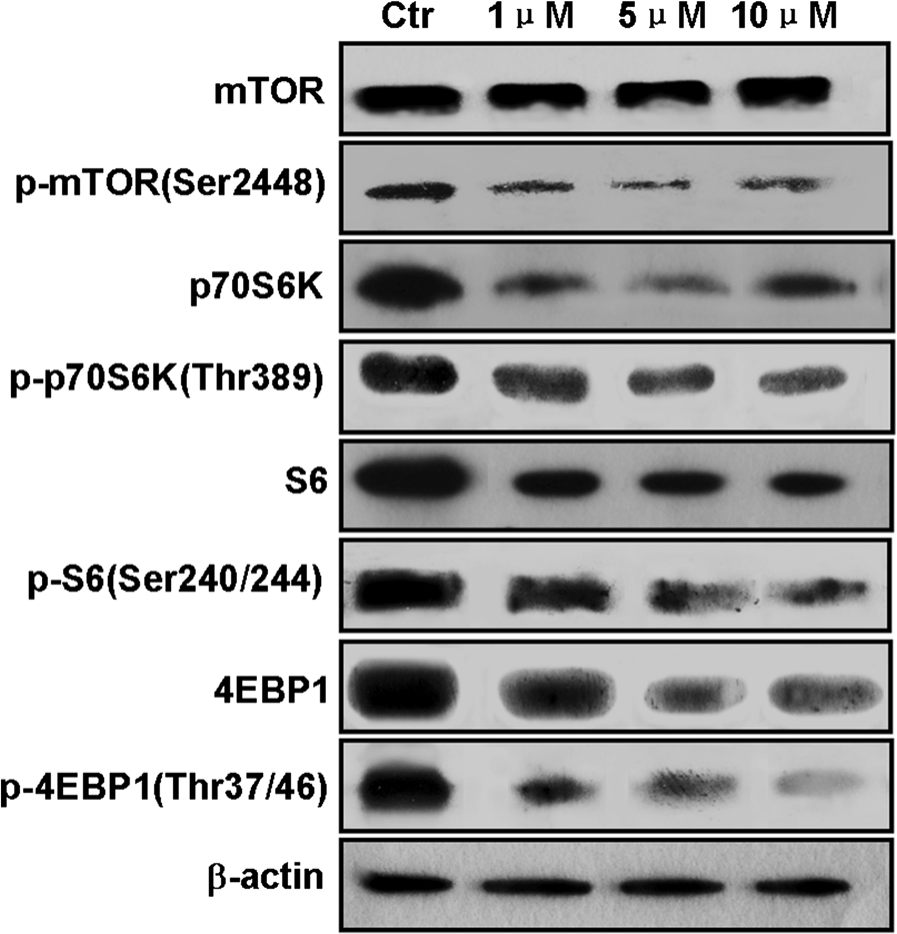
**Activation of mTOR and its downstream molecules is inhibited by CCI-779 in Bel-7402 cells.** Expression of mTOR, p70S6K, S6, and 4EBP1 and phosphorylation status of mTOR (Ser2448), p70S6K (Thr389), S6 (Ser240/244), and 4EBP1(Ser37/46) were examined by western blot 48 h after the addition of CCI-779. β-actin served as gel control.

To determine the mechanism of CCI-779 inhibition in Bel-7402 cells, we examined the activities of proteins in the mTOR signaling pathway by western blot. They were: mTOR and phospho-mTOR (Ser2448), downstream target p70S6K and p-p70S6K(Thr389), S6 and phospho-S6 (Ser240/244), 4EBP1 and p-4EBP1(Thr37/46). As shown in Figure 4, CCI-779 inhibited the phosphorylation of mTOR, p70S6K, S6 and 4EBP1, and slightly suppressed the expressions of mTOR, p70S6K, 4EBP1 and S6 in Bel-7402 cells.

### CCI-779 induced G_1_/S cell-cycle arrest in Bel-7402 cells

**Figure 5:**
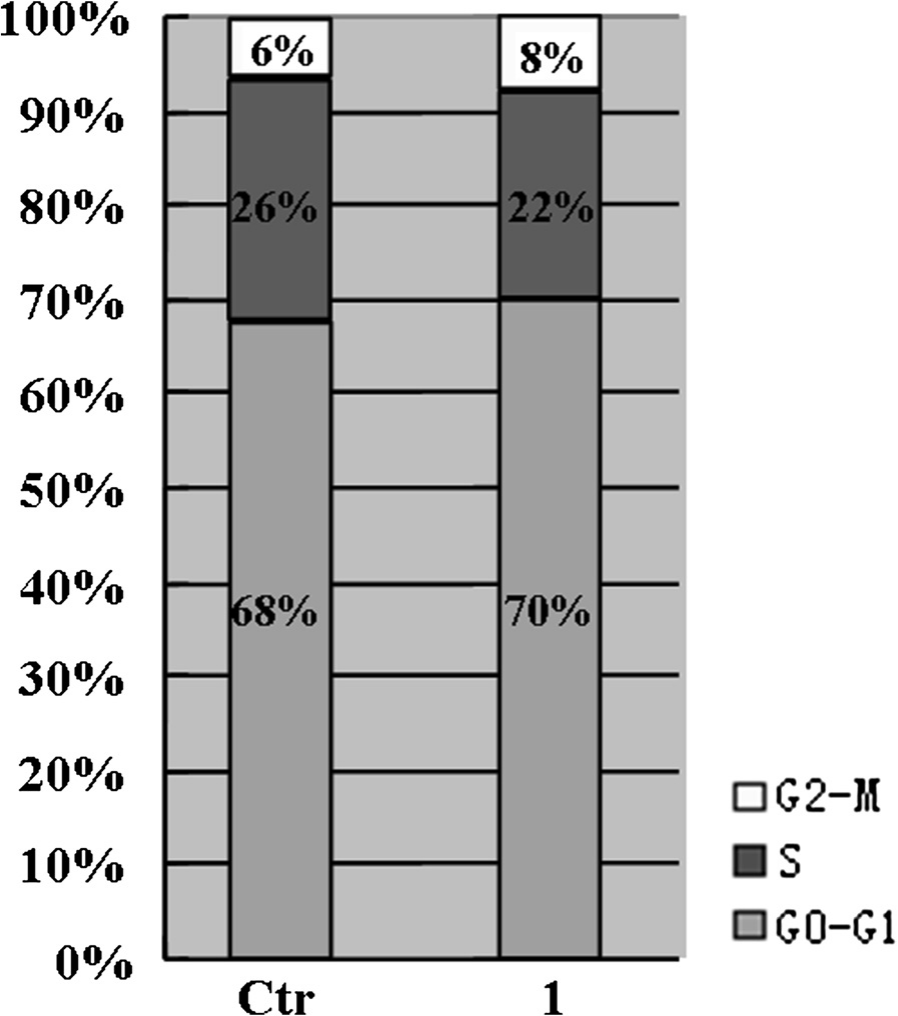
**CCI-779 induces G1/S cell cycle arrest in Bel-7402 cells.** CCI-779 treatment for 48 h and cell cycle analysis by flow cytometry. (Ctr), control without CCI-779; (1), treatment with 5 μM CCI-779 for 48 h.

To examine the effects of CCI-779 on cell cycle, Bel-7402 cells were incubated with 5 μM CCI-779 for 48 h and analyzed by flow cytometry. An inhibition of cell-cycle progression occurred as a result of CCI-779 treatment, as demonstrated by a decreased proportion of cells in S phase (Figure 5). These results indicate that CCI-779 can induce G1/S cell cycle arrest in Bel-7402 liver cancer cells.

### Morphological changes in Bel-7402 cells after CCI-779 treatment

**Figure 6:**
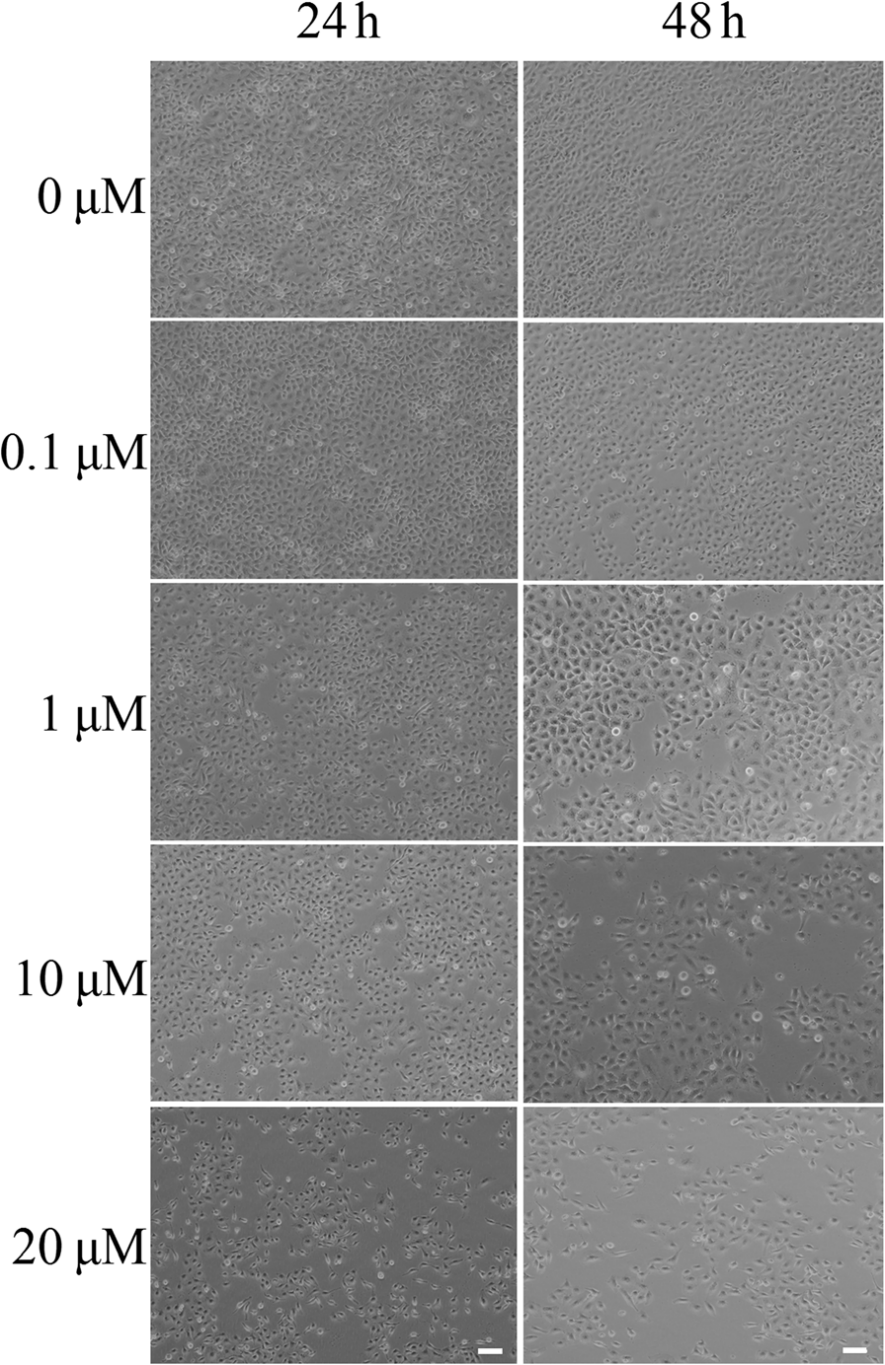
**CCI-779 induces changes in morphology of Bel-7402 cells.** Bel-7402 cells were treated with indicated concentrations of CCI-779, and microscopic images were taken at 24 h (**A**) and 48 h (**B**). Scale bar represents 50 micron.

mTOR is a central regulator of cell growth. With regard to lethality, we determined the effects of CCI-779 (0.1~20 μM) treatment for 24 and 48 h on Bel-7402 cells. At concentrations of CCI-779 ≥1 μM, cell density decreased and dead cells were observed (Figure 6).

Conventional therapeutic approaches for tumors include surgery, radiation, and chemotherapy. There has been much recent progress in the development of preventive and therapeutic strategies that target molecules in signal transduction pathways of cell proliferation, migration, and invasion in tumors [15-17]. Many specific inhibitors have been identified and are beginning to be used in clinical trials for cancer. CCI-779 has anti-proliferative effects against a variety of human tumor-derived cell lines [4,9,11,16,18] and has a good safety profile in clinical trails [12,13,19].

We examined the expression of mTOR in Bel-7402 liver cancer cells and HL-7702 normal liver cells by western blot. Consistent with previous data, mTOR levels were elevated in liver cancer cells versus normal liver cells. Further, 4EBP1, p70S6K, and S6 were also overexpressed. Rictor, which complexes with mTOR, is highly expressed in liver cancer [20]. These data indicate that the components of the mTOR pathway are upregulated in tumor cells.

Treatment of Bel-7402 cells with CCI-779 does not affect the phosphorylation of mTOR, p70S6K, or S6 after short (30 min) stimulations [4]. However, we observed that the phosphorylation of mTOR, p70S6K, 4EBP1 and S6 were inhibited after 48 h of treatment with CCI-779. Our data show that CCI-779 affects the activity of mTOR signaling pathway has a time-dependent manner in liver cancer cells. Meanwhile, the expression of p70S6K, 4EBP1 and S6 were inhibited after 48 h of treatment with CCI-779. Similar results also were found in our previous studies on esophageal tumor cells [21] and other groups [22,23]. The activity of S6 and 4EBP1 will decreased when mTOR was inhibited by its inhibitors, and then resulting in reduced protein synthesis, but the precise mechanism is unclear now. It is believed that the reduced expression of mTOR, 4EBP1 and S6 will inhibit cell proliferation.

Further, epigenetic mechanisms also govern the development of liver cancer. Several groups have found that histone deacetylase and microRNA mediate the pathogenesis of liver cancer [24,25]. These data demonstrate the importance of the mTOR signaling pathway in regulating cancer cell proliferation. Targeting proteins in the mTOR signal pathway might be a more effective approach in developing new drugs. For example, FKBP38 is a key regulator of mTOR. The binding of FKBP38 to mTOR inhibits the mTOR pathway; but Rheb can suppress FKBP38 and hence releases the growth signals [26]. These discoveries provide more targets in studying the inhibitors of the mTOR pathway and novel targets for cancer therapies.

mTOR has been proposed to regulate the basic process as a central regulator of cell growth [27], and there is a relationship between disorganization of the mTOR pathway and tumors [28,29]. In a previous study, we demonstrated that another clinical analog of rapamycin, RAD001, inhibits the growth of esophageal cancer cells in vitro [21]. Several studies have reviewed mTOR signaling in liver cancer and the occurrence of tumors, therapy, and the design of antitumor drugs [30,31]. The combination of two or more inhibitors has been proposed to enhance their effects on tumor suppression. Yet, attempts to combine two inhibitors [32] have not been efficacious. Recent years, transgenic mouse models in the development and progression of liver cancer was developed [33,34]. Undoubtedly, it will be a powerful tool that can be used to study liver cancer. Although rapamycin and its analogs are well established as anticancer drugs, new inhibitors of mTOR signaling must be identified and designed.

## Conclusions

In conclusion, our results demonstrate the importance of the mTOR signaling pathway in regulating liver cancer cell proliferation. CCI-779 has inhibitory effects on Bel-7402 liver cancer cells by suppressing the activity of mTOR and its downstream components. Thus, inhibition of mTOR is a potential therapeutic strategy for liver cancer.

## Materials and methods

### Cell lines and culture conditions

Bel-7402 human liver cancer cells were grown in 1640 medium, supplemented with 10% heat-inactivated fetal bovine serum. HL-7702 normal liver cells were maintained in 1640 medium, supplemented with 20% heat-inactivated fetal bovine serum. All cell lines were cultured in 5% CO_2_ at 37°C.

### Reagents

CCI-779 (temsirolimus), a derivative of rapamycin, was synthesized by Selleck Chemicals LLC (Huston, USA) and dissolved in DMSO (Sigma Chemical Corp., St. Louis, MO). The concentration of DMSO in the final solution did not exceed 1% (v/v).

### Trypan blue exclusion assay of cell proliferation

The antiproliferative effects of CCI-779 on Bel-7402 cells in culture were measured by trypan blue exclusion assay. Bel-7402 cells were used to seed 24-well culture plates at 1×10^4^ per well 24 h before drug treatment. For sensitivity experiments, subconfluent cells were treated with various concentrations CCI-779 (0.078 μM, 0.016 μM, 0.312 μM, 0.625 μM, 1.25 μM, 2.5 μM, and 5.0 μM) and 1% DMSO (v/v) for 48 h; harvested with trypsin; and stained with trypan blue. Cell number was counted using a hematocytometer.

For growth curve experiments, subconfluent cells were treated with various concentrations of CCI-779 (1 μM, 5 μM, 10 μM, and 20 μM) for 48 h, harvested with trypsin on the indicated day after treatment, and stained with trypan blue. Cell number was counted using a hematocytometer.

### MTT assay and IC_50_ calculation

Exponentially growing cells were used to seed 96-well plates at 4×10^3^ cells per well 48 h before drug treatment. Then, cells were incubated with CCI-779 at various concentrations (0.25 μM, 0.5 μM, 1.0 μM, 2.0 μM, 4.0 μM, 8.0 μM, 16.0 μM, 18.0 μM, 24.0 μM, and 28.0 μM) for 48 h. The medium with CCI-779 was absorbed, and fresh medium was added. 3-(4,5-dimethylthiazol-2-yl)-2,5-diphenyltetrazolium bromide (MTT, 5 g/l; Sigma-Aldrich, USA) was added to each well and incubated for 4 h at 37°C. The solution was absorbed, and formazan product was dissolved by adding 100 μl DMSO to each well and incubated it for 10 min at 37°C. MTT absorbance was measured at 490/630 nm with a spectrophotometer set (Thermo, Multiskan SX 353, USA). IC_50_ was calculated by Logit model, based on the data.

### Cell cycle analysis by flow cytometry

Bel-7402 cells were plated in 6-well tissue culture plates at 3×10^5^ cells per well and incubated for 48 h at 37°C. For cell cycle analysis, subconfluent cells were treated with 5 μM CCI-779 for 48 h and harvested. Cells were washed with cold PBS and stained with 50 mg/L propidium iodide (PI, Sigma-Aldrich, USA). DNA content was analyzed by flow cytometry (FACS Calibur, Becton-Dickinson Co., USA).

### Cell shape assay

Bel-7402 cells were used to seed 6-well culture plates at 3×10^5^ per well 24 h before drug treatment. Cells were treated with various concentrations of CCI-779 (0.1, 1, 10, and 20 μM) for 24 and 48 h and imaged with a digital camera that was mounted on a light microscope (Olympus DP-70, Japan).

### Western blot analysis

Bel-7402 cells were plated in 6-well plates at 3×10^5^ per well and incubated for 24 h in medium. On the following day, cells were treated with various concentrations of CCI-779 (1 μM, 5 μM and 10 μM) for 48 h and dissolved in cell lysis buffer (Thermo, USA). The concentrations of protein lysates were measured by Bio-Rad protein determination method (Bio-Rad, CA, USA).

Equal amounts (40 μg) of protein were electrophoresed in SDS-polyacrylamide gels, transferred to Immun-Blot™ PVDF membranes (Bio-RAD, CA, USA), and incubated with primary antibodies overnight at 4°C and peroxidase-linked secondary antibodies at room temperature for 1 h. Enhanced chemiluminescence (ECL) (GE Healthcare, UK) was used to detect the signals. The primary antibodies were against mTOR, phosphor-mTOR (Ser2448), phosphor-p70S6K (Thr389) (Abcam, UK); S6, phosphor-S6 (Ser240/244), phosphor-4EBP1 (Thr37/46) (Cell Signaling Technology, Inc., MA, USA); p70S6K, 4EBP1 (Santa Cruz Biotechnology, Inc., CA, USA), and β-actin (Sigma-Aldrich, USA).

### Statistical analysis

Descriptive statistics were generated for all quantitative data and expressed as mean ± SD. Proliferation of cells that were exposed to the drugs was compared with that of the negative control. Statistical significance was defined as *p < 0.05.

## Abbreviations

mTOR: Mammalian target of rapamycin; S6: Ribosomal protein S6; p70S6K: p70 ribosomal S6 kinase; 4EBP1: Eukaryotic translation initiation factor 4E-binding protein 1; IRS1: Insulin receptor substrate-1; PBS: Phosphate-buffered saline; SDS: Sodium dodecylsulfate

## Competing interests

The authors declare that they have no competing interests.

## Authors’ contributions

ZW, SL and YL conceived and designed the study. SL and YL drafted the manuscript. SL, YL, MW, XW, HF and YC performed the experimental studies. All authors have read and approved the final manuscript.
